# Transcriptome Analysis of *Arabidopsis thaliana* Plants Treated with a New Compound Natolen128, Enhancing Salt Stress Tolerance

**DOI:** 10.3390/plants10050978

**Published:** 2021-05-14

**Authors:** Kaori Sako, Chien Van Ha, Akihiro Matsui, Maho Tanaka, Ayato Sato, Motoaki Seki

**Affiliations:** 1 Department of Advanced Bioscience, Faculty of Agriculture, Kindai University, Nara 631-8505, Japan; 2 Plant Genomic Network Research Team, RIKEN Center for Sustainable Resource Science (CSRS), Yokohama 230-0045, Japan; chien.ha@ttu.edu (C.V.H.); akihiro.matsui@riken.jp (A.M.); maho.tanaka@riken.jp (M.T.); 3 Institute of Transformative Bio-Molecules (ITbM), Nagoya University, Nagoya 464-8601, Japan; ayato-sato@itbm.nagoya-u.ac.jp; 4 Kihara Institute for Biological Research, Yokohama City University, Yokohama 244-0813, Japan; 5 Plant Epigenome Regulation Laboratory, RIKEN Cluster for Pioneering Research, Wako, Saitama 351-0198, Japan

**Keywords:** salinity stress, chemical screening, nitric oxide

## Abstract

Salinity stress is a major threat to agriculture and global food security. Chemical priming is a promising approach to improving salinity stress tolerance in plants. To identify small molecules with the capacity to enhance salinity stress tolerance in plants, chemical screening was performed using *Arabidopsis thaliana*. We screened 6400 compounds from the Nagoya University Institute of Transformative Bio-Molecule (ITbM) chemical library and identified one compound, Natolen128, that enhanced salinity-stress tolerance. Furthermore, we isolated a negative compound of Natolen128, namely Necolen124, that did not enhance salinity stress tolerance, though it has a similar chemical structure to Natolen128. We conducted a transcriptomic analysis of Natolen128 and Necolen124 to investigate how Natolen128 enhances high-salinity stress tolerance. Our data indicated that the expression levels of 330 genes were upregulated by Natolen128 treatment compared with that of Necolen124. Treatment with Natolen128 increased expression of hypoxia-responsive genes including ethylene biosynthetic enzymes and *PHYTOGLOBIN*, which modulate accumulation of nitric oxide (NO) level. NO was slightly increased in plants treated with Natolen128. These results suggest that Natolen128 may regulate NO accumulation and thus, improve salinity stress tolerance in *A. thaliana*.

## 1. Introduction

More than 25% of irrigated land is affected by soil salinization worldwide [1]. High salinity stress is one of the major limiting factors of crop productivity and growth. Salt stress causes osmotic stress, ionic stress and oxidative stress [2,3]. A high concentration of salt makes plants harder to absorb water and nutrients and an ion imbalance prevents plant growth by altering metabolic processes and reducing photosynthesis [2]. Under salinity-stress conditions, reactive oxygen and nitrogen species (ROS and RNS, respectively) are also produced in plant cells. Low levels of ROS and RNS work as messengers of signal transduction [4]. For example, nitric oxide (NO) belonging to the RNS family modulates S-nitrosylation and regulates abiotic stress responses. A recent report showed that NO regulates the activity of Protein arginine methyltransferases 5 (PRMT5), which catalyzes the symmetric demethylation of protein arginine residues under salinity stress, by S-nitrosylation, resulting in the proper stress response [5]. However, excessive ROS and RNS accumulation can lead to cellular oxidative damage [6]. Thus, plants are equipped with mechanisms to control the amount of ROS and RNS such as enzymatic and non-enzymatic antioxidant defense systems. Enzymatic antioxidants include Superoxide dismutase, Catalase, Ascorbate peroxidase and glutathione peroxidase [7]. Non-enzymatic antioxidants such as anthocyanin and ascorbate have antioxidant activity [8].

As the world’s population is estimated to reach 9.7 billion by 2050 [9], technological development to improve crop salinity-stress tolerance is crucial for sustainable food production. Latest research suggests that chemical priming may improve abiotic stress tolerance in crops [10]. Chemical priming is an approach in which plants are treated with natural or synthetic compounds prior to stress exposure. Treated plants exhibit greater stress tolerance compared with non-treated plants, namely primed state. This method has the advantage that chemical priming agents often improve abiotic stress tolerance in non-specific plant species, so it may be applied to various plant species. Previously, we isolated a novel chemical agent enhancing high-salinity stress tolerance in Arabidopsis and rice from the RIKEN chemical library [11]. This study indicated that chemical libraries might include valuable compounds to improve abiotic stress tolerance in various agricultural crops. We therefore screened compounds enhancing high-salinity stress tolerance using the Institute of Transformative Bio-Molecule (ITbM) chemical library. We found that NaCl tolerance enhancer128 (Natolen128) improved salinity stress tolerance in *Arabidopsis thaliana* at a low concentration. We conducted a transcriptome analysis to clarify the mechanism enhancing high-salinity stress tolerance. The transcriptomic analysis revealed that the expression levels of abiotic stress responsive genes, including ethylene biosynthetic genes (such as *AtACS9*) and hypoxia-responsive genes (such as *AtPGB1*), were upregulated by Natolen128.

## 2. Results

### 2.1. Natolen128 Was Identified as a New Compound Enhancing Salt Stress Tolerance in A. thaliana

**Figure 1:**
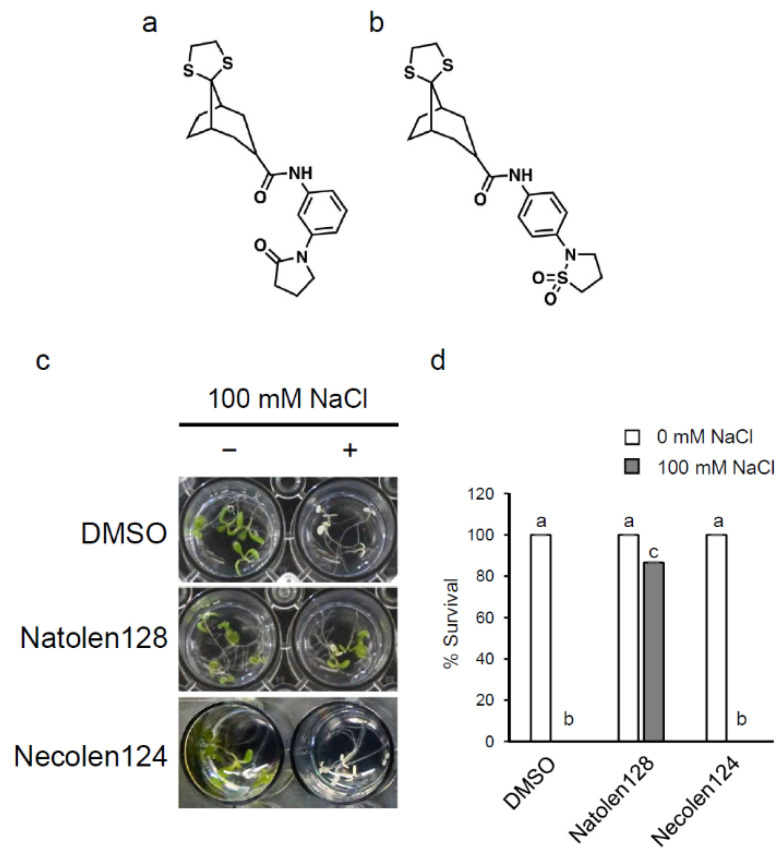
Natolen128 enhances high-salinity stress tolerance in *Arabidopsis thaliana*. (**a**) Structure of Natolen128. (**b**) Structure of Necolen124. (**c**) Morphology of seedlings treated with 2 µM Natolen128, with or without subsequent treatment of 100 mM NaCl for 4 days. Necolen124 and DMSO were used as negative controls. The inside diameter of each well is 15.4 mm. (**d**) Survival rate under high-salinity conditions in the presence or absence of 2 µM Natolen128. The survival rate of 15 plants was calculated 4 days after NaCl treatment. These experiments were conducted using three biological replicates. Error bars represent the mean ± standard error (SE). Statistical significance was determined by ANOVA, followed by post-hoc Tukey’s tests. Means that differed significantly (*p* < 0.05) are indicated by different letters.

To identify compounds that enhance salt-stress tolerance in plants, we screened 6400 compounds from the ITbM library. *Arabidopsis* wild-type Col-0 plants grown in liquid culture medium for 4 days were treated with each library compound for 24 h, then subsequently treated with 100 mM NaCl. We investigated the survival rates for 4 days and identified four compounds that showed higher survival rates under high salt-stress conditions. Among them, we focused on *N*-[3-(2-oxo-1-pyrrolidinyl)phenyl]-spiro[bicyclo[3.2.1]octane-8,2′-[1,3]dithiolane]-3-carboxamide (Natolen128) (Figure 1a), because it showed the strongest increase in tolerance to salinity stress. To investigate the function of Natolen128, we next screened negative compounds of Natolen128 that had similar chemical structures but did not enhance salt stress tolerance. We evaluated five candidate compounds and isolated *N*-[4-(1,1-dioxido-2-isothiazolidinyl)phenyl]-spiro[bicyclo[3.2.1]octane-8,2′-[1,3]dithiolane]-3-carboxamide as a negative compound of Natolen128 (Necolen124) (Figure 1b). To confirm the salinity-stress tolerance increase from Natolen128, wild-type plants grown in liquid culture medium for 4 days were treated with 2 µM Natolen128, Necolen124 or DMSO for 24 h, with or without subsequent treatment with 100 mM NaCl for 4 days. The plants treated with Natolen128 showed an increased survival rate under salinity-stress conditions compared with DMSO-treated and Necolen124-treated plants (Figure 1c,d).

### 2.2. Comparative Transcriptomic Analysis of Natolen128 Treatments under High-Salinity Stress Conditions

**Figure 2:**
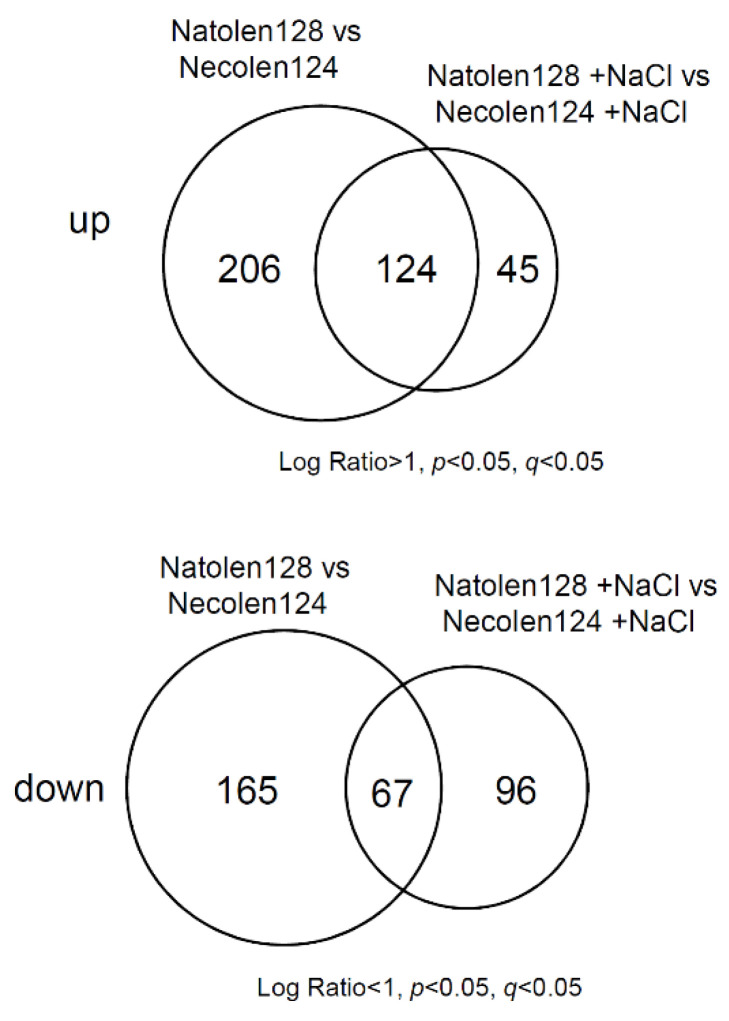
Gene expression profiles under Natolen128 and salinity stress treatments. The Venn diagrams show genes exhibiting up- and down-regulated expression in response to Natolen128 and Necolen124 treatments under control and high-salinity stress conditions. Each treatment was performed using 20 plants, with three biological replicates.

To elucidate the molecular mechanism of high salt-stress tolerance mediated by Natolen128, we conducted global gene expression analysis with a microarray. Four-day-old plants treated with 2 µM Natolen128 or Necolen124 for 24 h, with or without a subsequent treatment with 100 mM NaCl for 2 h, were examined. We identified 330 genes whose expression was increased by 24 h Natolen128 treatment compared with Necolen124 (Table S1) and 232 genes whose expression was decreased by 24 h Natolen128 treatment compared with Necolen124 (Table S2). Additionally, 169 genes were more highly expressed in Natolen128-treated plants than in Necolen124-treated plants under high-salinity stress conditions (Table S3), whereas 163 genes showed lower expression in Natolen128-treated plants under salinity stress conditions than in Necolen124-treated plants (Table S4). Venn diagrams prepared based on the microarray data indicated that 124 overlapping genes exhibited higher expression and 67 overlapping genes exhibited lower expression in Natolen128-treated plants than in Necolen124-treated plants under control and high-salinity stress conditions (Figure 2, Table S5).

### 2.3. Natolen128 Induces the Expression of a Phytoglobin and Ethylene Biosynthetic Genes under High-Salinity Stress Conditions

**Figure 3:**
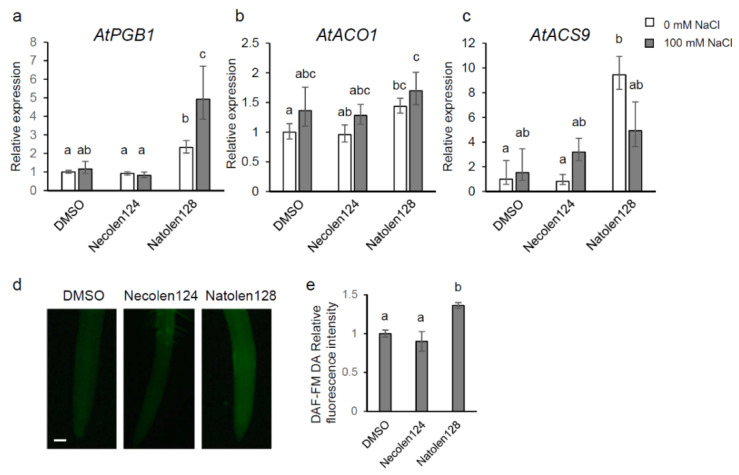
Hypoxia-responsive gene expression and NO changes due to Natolen128 treatment. (**a**–**c**) Relative expression levels of the *AtPGB1* (**a**), *AtACO1* (**b**) and *AtACS9* (**c**) genes during salinity-stress treatment for 0 and 2 h with or without 2 µM Natolen128 or 2 µM Necolen124. The expression level in plants treated with DMSO was set as 1. *18S rRNA* was used as an internal standard. Error bars represent the mean ± SE (*n* = 3). Statistical significance was determined by ANOVA, followed by post-hoc Tukey’s tests. Means that differed significantly (*p* < 0.05) are indicated by different letters. (**d**) NO detected with DAF-FM DA in Arabidopsis roots treated with 2 µM Natolen128 or Necolen124 for 6 h. Plants treated with DMSO for 6 h were used as negative controls. Scale bar = 50 µm. (**e**) Relative fluorescence intensity of DAF-FM DA in roots treated with 2 µM Natolen128 or 2 µM Necolen124. The fluorescence intensity in plants treated with DMSO was set as 1. Statistical significance was determined by ANOVA, followed by post-hoc Tukey’s tests. Means that differed significantly (*p* < 0.05) are indicated by different letters.

We focused on the 124 genes upregulated by Natolen128 both before and after high-salinity stress conditions. To unravel the function of Natolen128, GO analysis of these 124 genes was performed. GO analysis indicated that 29 genes were categorized as stress response-related (Table 1). We focused particularly on *Hemoglobin/Phytoglobin 1* (*AtPGB1*) and *ACC OXIDASE 1* (*AtACO1*), because these genes have been reported to respond to salinity stress [12,13,14]. AtPGB1 is a non-symbiotic class I hemoglobin that modulates the NO concentration [15]. AtACO1 is an ethylene biosynthetic enzyme catalyzing the conversion of 1-aminocyclopropane-1-carboxylic acid (ACC) to ethylene. A qRT-PCR assay was performed to validate the expression of these candidate genes. *AtPGB1* expression was upregulated in Natolen128-treated plants compared with DMSO- and Necolen124-treated plants under control conditions. Further, the expression of *AtPGB1* was significantly increased by Natolen128 treatment under high-salinity conditions (Figure 3a). *AtACO1* expression was slightly increased compared with DMSO-treated plants under control conditions (Figure 3b). Furthermore, Natolen128 treatment induced the expression of an ethylene receptor (*AtETR2*) and an ethylene response transcription factor (*AtHRE2*) (Table 1). Therefore, we investigated the expression of *1-AMINOCYCLOPROPANE-1-CARBOXYLATE SYNTHASE 9* (*AtACS9*), which encodes an ethylene biosynthetic enzyme, and found it was upregulated by Natolen128 treatment under control conditions (Figure 3c), suggesting that ethylene biosynthesis was activated by Natolen128 treatment. Ethylene and AtPGB1 has been reported to regulate the intracellular NO content [16]. Thus, we observed the NO content in roots using a NO probe, DAF-FM DA. A slight NO signal was detected in roots treated with Natolen128 for 6 h, but there was no signal in DMSO-treated roots (Figure 3d,e). These results implied that Natolen128 might induce NO production and gene expression of *AtPGB1* and ethylene biosynthetic enzymes, leading to the high-salinity stress tolerance phenotype.

## 3. Discussion

In this study, we identified a compound, Natolen128, that enhances salinity stress tolerance in *Arabidopsis thaliana* by screening the ITbM chemical library. Additionally, Necolen124 was isolated as a negative compound of Natolen128 that has a similar structure but does not enhance salinity stress tolerance. Transcriptome analysis showed that Natolen128 increased the expression of *AtPGB1* and ethylene biosynthetic enzyme genes. Moreover, the NO content was slightly increased by Natolen128.

The plant hormone ethylene plays an important role in abiotic stress responses, and the application of exogenous ethylene and ACC improves salt tolerance in *Arabidopsis* [17,18], showing that ethylene contributes to salt stress tolerance in plants. Ethylene biosynthesis is characterized by two steps; first, the substrate S-adenosyl-l-methionine is converted to ACC by ACS [19,20]. The Arabidopsis genome contains eight functional ACS genes (*ACS2*, *ACS4–9* and *ACS11*) and each of these genes contributes to ethylene production [21]. In the second step, ACC is converted to ethylene by ACO [22,23]. ACS and ACO mainly regulate the ethylene biosynthesis pathway. The expression levels of *AtACO1* and *AtACS9* were increased by Natolen128 treatment under control conditions. These results suggest that Natolen128 activates ethylene biosynthesis. Ethylene signaling has been shown to modulate salinity stress responses via maintaining Na^+^/K^+^ and ROS homeostasis and regulating microtubule reassembly [24,25]. Thus, ethylene produced by Natolen128 treatment might induce primed state and rapidly activate the stress response under salinity stress condition leading to enhance tolerance.

In addition, ethylene is produced under early hypoxia stress and enhances acclimation to hypoxia [26]. Previous reports showed that ethylene induces *AtPGB1* expression during hypoxia and AtPGB1 participates in the low-oxygen stress response by detoxifying NO [16,27]. We showed that *AtPGB1* was highly expressed in plants treated with Natolen128 under salinity stress. Interestingly, in addition to *AtPGB1*, Natolen128 induced many hypoxia-responsive genes, such as *HYPOXIA RESPONSIVE ERF2 (AtHRE2)*, *LOB domain-containing protein41 (AtLBD41)*, *PYRUVATE DECARBOXYLASE 1 (AtPDC1)*, *HYPOXIA RESPONSE ATTENUATOR1 (AtHRA1)* and the cysteine oxidases *AtPCO1* and *AtPCO2* [28]. These results imply that Natolen128 treatment may induce a low O_2_-like condition in plants. The emission of NO is increased in plants under low O_2_ conditions [29]. Thus, we investigated NO levels in roots treated with Natolen128 and we detected slightly increased NO levels in roots treated with Natolen128. NO plays an essential role in plant abiotic stress responses. Treatment with sodium nitroprusside (SNP), a donor for NO, enhanced salinity stress tolerance [30] and we confirmed that SNP enhanced salinity stress tolerance in our growth conditions (Figure S1). Thus, we assume that Natolen128 activates NO production under control conditions and this NO works as a signaling molecule inducing acclimation to salinity stress in plants. In addition, NO induced by Natolen128 treatment presumably induces hypoxia responsive genes. However, an excess amount of NO is harmful to cells [31]. We observed that the expression of *AtPGB1* was increased by Natolen128 treatment under high-salinity conditions, suggesting that AtPGB1 may remove excess NO in Natolen128-treated plants under salinity stress conditions. Our findings suggest that Natolen128 regulates intracellular NO homeostasis, resulting in enhanced salt stress tolerance in *Arabidopsis thaliana*.

## 4. Materials and Methods

### 4.1. Plant Materials and Growth Conditions

*A. thaliana* (ecotype Columbia-0) seeds were sterilized and sown in half-strength Murashige and Skoog (MS) liquid medium supplemented with 1% sucrose and 0.1% agar. The plants were grown under previously described conditions [32]. Four-day-old plants were treated with compounds for 24 h, with or without subsequent treatment with 100 mM NaCl (WAKO, Japan). The NaCl solution was added into the medium containing both the compounds and plants. The survival rate of 20 plants was calculated 4 days after the NaCl treatment. The experiment was conducted using three biological replicates.

### 4.2. Chemical Library Screening

A chemical library (10 mM of each compound in dimethylsulfoxide (DMSO)) was obtained from the ITbM library. We sowed 5–6 Col-0 seeds in each well containing 250 µL of half-strength MS liquid medium in a 96-well plate and the seeds were grown for 4 days. Subsequently, 1 µM chemical (final concentration) was added to each well and incubated for 24 h, and the plants were then treated with 100 mM NaCl. Survival rates were checked 4 days after the NaCl treatment.

### 4.3. RNA Extraction

Total RNA was extracted from 5-day-old *A. thaliana* seedlings treated with 2 µM Natolen128 for 24 h, with or without subsequent treatment with 100 mM NaCl for 2 h. DMSO and Necolen124 were used as negative controls. For qRT-PCR, total RNA was extracted from 5–10 plants using the Plant RNA reagent (Thermo Fisher Scientific, Waltham, MA, USA) as previously described [33]. For microarray analysis, total RNA was extracted from 20 plants with an RNeasy Plant Mini Kit (Qiagen, Hilden, Germany) according to the manufacturer’s instructions. The quality of the extracted total RNA was evaluated using a Bioanalyzer system (Agilent, Santa Clara, CA, USA). The experiment was performed using three biological replicates.

### 4.4. Microarray Analysis

A microarray analysis was completed as previously described [11]. The microarray data were deposited in the GEO database (GEO ID: GSE173340). Each treatment was analyzed using three biological replicates, and 10 plants were sampled for each treatment and/or repeat. Genes with an expression log_2_ ratio ≥ 1 [*t*-test analysis, Benjamini–Hochberg correction (FDR) < 0.05] were identified as upregulated genes. Gene Ontology (GO) assignments for *Arabidopsis* genes were obtained from TAIR (www.arabidopsis.org, last accessed on 12 May 2021).

### 4.5. Quantitative Real-Time PCR Analysis

First-strand cDNA synthesis was performed with a PrimeScript™ RT reagent Kit (Takara, Kusatsu Japan) for quantitative real-time polymerase chain reaction (qRT-PCR) analysis. The qRT-PCR was conducted as previously described [11] and *18S rRNA* was used as a reference gene. The experiment was conducted using three biological replicates and 10 plants were sampled for each treatment and/or repeat. The qRT-PCR primer sequences were as follows: *AtPGB1*, 5′-AACACTTTGAGGTGGCCAAG-3′ and 5′-TGATCATAAGCCTGACCCCAAG-3′; *AtACS9*, 5′-AAATGGAGAACGGGAGCAGA-3′ and 5′-AAGAGGGTTAGACGGGTTGG-3′; *AtACO1*, 5′-GATCAAAGAGAGAGAGATGGAGA-3′ and 5′-TGAAATGTTTGGGATCTGACAGAT-3′; *At18S rRNA*, 5′-CGGCTACCACATCCAAGGAA-3′ and 5′-GCTGGAATTACCGCGGCT-3′.

### 4.6. NO Detection

Intracellular NO levels were visualized using DAF-FM DA (diaminofluorescein-FM diacetate, Goryo Chemical) as previously described [16]. Seedlings treated with 2 µM Natolen128 or DMSO for 6 h were incubated in the dark for 15 min under gentle agitation in 10 mM Tris-HCl buffer (pH 7.4) containing 10 μM DAF-FM DA and subsequently washed twice for 5 min with 10 mM Tris-HCl buffer (pH 7.4). Fluorescence was visualized using an Olympus BX51 fluorescence microscope. Fluorescence intensity in root tips was determined by ImageJ software (https://imagej.nih.gov/ij/index.html, last accessed on 12 May 2021).

## Figures and Tables

**Table 1:** List of Natolen128-upregulated genes classified as stress responsive ^(1)^.

Gene Name	AGI Code	Natolen128/Necolen124 under Control Condition			Natolen128 under Salt-stress/Necolen124 under Salt-Stress		
		Ratio ^(2)^	*p*-Value	FDR	Ratio ^(3)^	*p*-Value	FDR
*Germin-like Protein 7 (GLP7)*	AT1G10460	1.753	0.001	0.005	1.208	0.004	0.029
*HEAVY METAL ASSOCIATED PROTEIN 4 (HMP04)*	AT1G22990	1.382	0.000	0.004	1.572	0.004	0.030
*LOW PHOSPHATE ROOT1 (LPR1)*	AT1G23010	1.511	0.000	0.004	1.223	0.001	0.020
*ACYL-ACYL CARRIER PROTEIN 6 (AAD6)*	AT1G43800	2.045	0.000	0.002	3.673	0.000	0.016
*Cytochrome P450 superfamily protein*	AT1G66540	1.072	0.001	0.006	1.232	0.002	0.023
*Hemoglobin/phytoglobin1 (PGB1)*	AT2G16060	2.415	0.000	0.003	2.304	0.001	0.020
*ACC oxidase 1 (ACO1)*	AT2G19590	1.646	0.005	0.012	1.469	0.000	0.010
*DROUGHT TOLERANCE REPRESSOR (DOR)*	AT2G31470	1.238	0.003	0.010	1.205	0.001	0.019
*Peroxidase superfamily protein*	AT2G35380	1.204	0.005	0.013	1.049	0.001	0.021
*Peroxidase superfamily protein*	AT2G38390	1.828	0.000	0.003	1.318	0.000	0.011
*Hypoxia Responsive ERF2 (HRE2)*	AT2G47520	2.578	0.000	0.004	1.457	0.006	0.034
*LOB domain-containing protein 41 (LBD41)*	AT3G02550	3.524	0.000	0.005	4.451	0.000	0.010
*GAST1 protein homolog 5 (GASA5)*	AT3G02885	1.623	0.009	0.016	2.275	0.000	0.009
*Haloacid dehalogenase-like hydrolase (HAD) superfamily protein*	AT3G19595	1.622	0.002	0.008	1.460	0.001	0.021
*Ethylene receptor 2 (ETR2)*	AT3G23150	1.419	0.000	0.005	1.015	0.004	0.030
*HYPOXIA RESPONSE UNKNOWN PROTEIN 6 (HUP6)*	AT3G27220	1.926	0.001	0.007	3.014	0.000	0.017
*Sucrose synthase 4 (SUS4)*	AT3G43190	1.624	0.013	0.020	1.888	0.013	0.045
*FATTY ACID REDUCTASE 4 (FAR4)*	AT3G44540	1.812	0.001	0.006	1.062	0.003	0.027
*METHYL ESTERASE 10 (MES10)*	AT3G50440	1.759	0.000	0.003	1.082	0.003	0.027
*EARLY LIGHT-INDUCIBLE PROTEIN 2 (ELIP2)*	AT4G14690	1.036	0.000	0.005	1.150	0.000	0.009
*Unknown protein*	AT4G17670	1.613	0.000	0.005	1.463	0.001	0.021
*ABC1-LIKE KINASE 1 (ABC1K1)*	AT4G31390	1.767	0.001	0.006	1.186	0.000	0.011
*GLUTATHIONE PEROXIDASE 7 (GPX7)*	AT4G31870	1.921	0.001	0.006	1.883	0.002	0.022
*Hemoglobin/Phytoglobin 3 (PGB3)*	AT4G32690	1.152	0.000	0.003	1.122	0.001	0.019
*Pyruvate decarboxylase 1 (PDC1)*	AT4G33070	2.324	0.000	0.004	1.944	0.011	0.043
*WOUND-INDUCED POLYPEPTIDE 5 (WIP5)*	AT4G33560	3.159	0.002	0.009	5.124	0.000	0.011
*Plant cysteine oxidase 1 (PCO1)*	AT5G15120	2.655	0.001	0.006	3.614	0.000	0.009
*Plant cysteine oxidase 2 (PCO2)*	AT5G39890	3.245	0.000	0.004	4.733	0.000	0.014
*DEFECTIVE IN INDUCED RESISTANCE 1 (DIR1)*	AT5G48485	1.105	0.006	0.014	1.049	0.000	0.009

^(1)^ The genes were selected from 124 overlapping genes using two conditions: (a) log_2_ ratio (plants treated with Natolen128 for 24 h/plants treated with Necolen124 for 24 h) ≥ 1, FDR < 0.05, *t*-test < 0.05; (b) log_2_ ratio (plants treated with Natolen128 for 24 h followed by 2 h NaCl treatment/plants treated with Necolen124 for 24 h followed by 2 h NaCl treatment) ≥ 1, FDR < 0.05, *t*-test < 0.05. ^(2)^ The values represent the log_2_ ratio (plants treated with Natolen128 for 24 h/plants treated with Necolen124 for 24 h). ^(3)^ The values represent the log_2_ ratio (plants treated with Natolen128 for 24 h followed by 2 h NaCl treatment/plants treated with Necolen124 for 24 h followed by 2 h NaCl treatment).

## Data Availability

The data presented in this study are available in the article and in the Supplementary Materials.

## Supplementary Materials

The following are available online at https://www.mdpi.com/article/10.3390/plants10050978/s1, Figure S1: SNP enhances high-salinity stress tolerance in *Arabidopsis thaliana*, Table S1: List of the genes that were up-regulated by Natolen128 compared with Necolen124 treatment under control condition, Table S2: List of the genes that were down-regulated by Natolen128 compared with Necolen124 treatment under control condition, Table S3: List of the genes that were up-regulated by Natolen128 compared with Necolen124 treatment under salt stress, Table S4: List of the genes that were down-regulated by Natolen128 compared with Necolen124 treatment under salt stress, Table S5: List of overlapping genes that were up-regulated by Natolen128 compared with Necolen124 treatment both under control and salinity stress condition.
