# Supplementary material for: Transcriptome Analysis of Arabidopsis thaliana Plants Treated with a New Compound Natolen128, Enhancing Salt Stress Tolerance
Source: Plants (Basel). 2021 May 14;10(5):978. doi: 10.3390/plants10050978 (PMC8153642; doi:10.3390/plants10050978)
Supplement: Supplementary file 1 [file plants-10-00978-s001.zip › plants-1218322-supplementary/Figure S1.pdf]

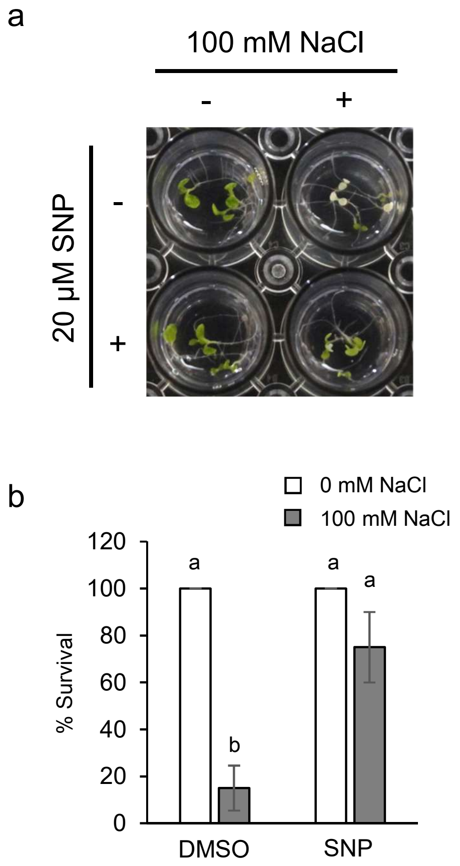

Supplemental Figure 1 SNP enhances high-salinity stress tolerance in Arabidopsis thaliana. (a) Morphology of seedlings treated with 20 μM SNP, with or without subsequent treatment of 100 mM NaCl for 4 days. DMSO was used as negative controls. The inside diameter of each well is 15.4 mm. (b) Survival rate under high-salinity conditions in the presence or absence of 20 μM SNP. The survival rate of 15 plants was calculated 4 days after NaCl treatment. These experiments were conducted using three biological replicates. Error bars represent the mean ± standard error (SE). Statistical significance was determined by ANOVA, followed by post-hoc Tukey's tests. Means that differed significantly (p < 0.05) are indicated by different letters.
